# Voltage-Gated Sodium Channel Na_V_1.2: Structural Perspective of the Genetic Variability

**DOI:** 10.1007/s10528-026-11327-z

**Published:** 2026-02-03

**Authors:** Tomás Oliveira-Madureira, Bárbara Leal, Luísa Azevedo

**Affiliations:** 1 Unit for Multidisciplinary Research in Biomedicine (UMIB), School of Medicine and Biomedical Sciences (ICBAS), University of Porto, Rua Jorge Viterbo Ferreira 228, 4050-313 Porto, Portugal; 2 ITR - Laboratory for Integrative and Translational Research in Population Health, Rua das Taipas 135, 4050-600 Porto, Portugal

**Keywords:** Voltage-gated sodium channel, Na_v_1.2, Epilepsy, Autism spectrum disorders, Genetics, Structural bioinformatics

## Abstract

The *SCN2A* gene encodes the alpha subunit of a voltage-gated sodium channel which is necessary for creating and propagating action potentials in neurons. Impairment of the Na_v_1.2 function is associated with neurodevelopmental disorders such as Developmental and Epileptic Encephalopathy, Self-limited Neonatal/Infantile Seizures, and Autism Spectrum Disorder. In this study, we used orthologous sequence comparisons, as well as disease associated variants to analyze their location in the structure of Na_v_1.2, with the aim of understanding the impact of genetic variants in the structure of this protein at an evolutionary and clinical level. Our analyses reveal different spatial distribution of interspecific variation where different residues locate preferentially in the first cytoplasmic linker, while disease-associated variants tend to cluster in the voltage-sensing segments of the protein domains. Altogether, these discoveries point to structurally important segments in the Na_v_1.2 structure that have been conserved through evolution due to their role in maintaining the function of the channel.

**Supplementary Information:**

The online version contains supplementary material available at 10.1007/s10528-026-11327-z.

## Introduction

The SCN (Voltage-gated sodium channel) gene family has 10 members, from *SCN1A* to *SCN11A*, which are necessary for the generation and propagation of action potentials in neurons and muscles (Wang et al. 2017). In humans, the sodium voltage-gated channel alpha subunit 2 gene *(SCN2A)* encodes the neuronal voltage-gated sodium channel Na_v_1.2. The *SCN2A* gene contains 27 exons and encodes a protein of 2005 amino acids (Kasai et al. 2001). Two isoforms associated with this transcript differ in one amino acid residue at position 209. These two isoforms have different developmental roles: the “neonatal” isoform Asn209 (5 N) is found in early development in cortical neurons, while the “adult” isoform which contains Asp at the same position (5 A) replaces the former after birth (Liang et al. 2021; Sanders et al. 2018).

Na_v_1.2 contains four domains labeled as domains I to IV. Each domain contains six membrane segments (S1 to S6). The S1-S4 segments are voltage-sensing domains, while the S5-S6 segments form the pore loops and DEKA (aspartate-glutamate-lysine-alanine) motif selectivity filters (Lipkind and Fozzard 2008), respectively. The S4 voltage sensing segment is necessary to sense the charge differences between the intracellular and extracellular sides of the membrane (de Lera Ruiz and Kraus 2015). When the electrical difference between the intra and extracellular sides becomes negative, the voltage-sensing domains suffer conformational changes that in turn lead to the activation of the channel. Sequentially, the central ion selectivity pore opens, allowing sodium influx into the intracellular space. Subsequently, a conformational change occurs where the pore is physically blocked by the inactivation gate on the intracellular side of the cell. Finally, the inactivation gate is then released by the subsequent hyperpolarization of the cell, allowing for its recovery (Payandeh et al. 2011, 2012; McCusker et al. 2012).

*SCN2A* deleterious variants have been mainly associated with Developmental and Epileptic Encephalopathy (DEE) (Ogiwara et al. 2009; Kodera et al. 2013; Sundaram et al. 2013; Saitoh et al. 2015; Ben-Shalom et al. 2017), Self-limited Neonatal/Infantile seizures (SeLNE) (Sugawara et al. 2001; Heron et al. 2002; Herlenius et al. 2007), and Autism Spectrum Disorders (ASD)/Intellectual Disability (ID) (Sanders et al. 2018; Lopes-Marques et al. 2024). Loss-of-function variants that lower Na_v_1.2 activity are seen in patients with ASD, while gain-of-function variants, those that increase Na_v_1.2 activity, are associated with SeLNE (Scalmani et al. 2006; Ogiwara et al. 2009; Rauch et al. 2012; Lauxmann et al. 2013; Schwarz et al. 2016; Ben-Shalom et al. 2017; Sanders et al. 2018; Wang et al. 2021).

Here, we study the different types of missense variants found in *SCN2A* from both the evolutionary and clinical context. We analysed the 2D and 3D structure of the Na_v_1.2 protein, recognizing mutational hotspots that differ between the two contexts and inferring about the spatial clustering of the amino acid residues. This information is important in the evaluation of the functional effect of new variants and for a deeper understanding of individual variability in disease presentation.

## Materials and Methods

### Na_v_1.2 Sequence Alignment

Since the focus of this work is related to missense variants, we started by analysing and comparing the sequences of the Na_v_1.2 protein between humans and non-human primates (chimpanzee, gorilla, rhesus monkey, and orangutan) and rodents (mouse and rat). The Ensembl database (release 112) (Harrison et al. 2024) was used to extract the orthologous sequence of Na_v_1.2 in these species. ClustalOmega was used for the alignment of these protein sequences (Madeira et al. 2024).

### Na_v_1.2 Conservation Scores

Grantham (Grantham 1974) scores were determined for inter species differences, allowing us to examine further the degree of conservation of the Na_v_1.2 protein between humans and non-human mammals. For amino acid substitutions, Grantham scores were classified as conservative (score: 0–50) moderately conservative (score: 51–100), moderately radical (score: 101–150), and radical (score > 151) (Li et al. 1984). Additionally, the ConSurf web server (consurf.tau.ac.il/) was used to access the conservation profile of the Na_v_1.2 protein. Each amino acid position is scored from 1 (least conserved) to 9 (most conserved), relative to the degree of evolutionary conservation (Ashkenazy et al. 2016).Variant Database The gnomAD database (Karczewski et al. 2020) was used to access the variability spectrum of the *SCN2A* gene data extracted on 12/09/2024.

### Structural Analyses of Na_v_1.2

For the structural analyses of Na_v_1.2 we used the ClinVar (Landrum et al. 2014) database to retrieve *SCN2A* missense variants filtered as “pathogenic” in the germline classification and as “missense” in the molecular consequence filter. From this set we extracted the variants associated with DEE and SeLNE resulting in a total of 91 variants. The ASD-associated variants were obtained from the work of Zhou et al. 2022 and Sanders et al. 2018. This list includes a total of 46 variants (Supplementary file 1).

**Fig. 1:**
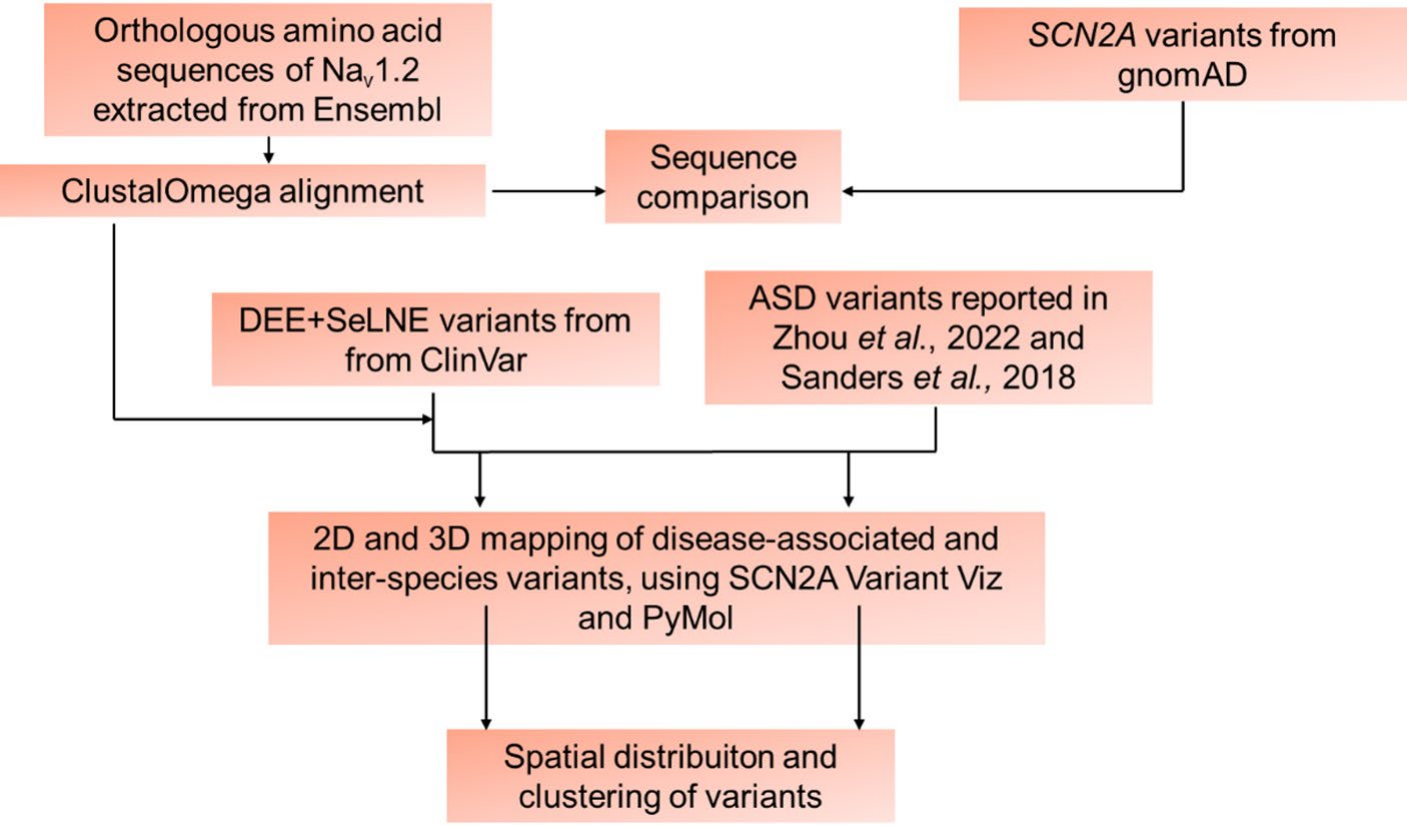
Methodology used in this work

The position of variants in the structure of Na_v_1.2 was performed according to SCN2A Variant Viz 6.0 (https://public.tableau.com/app/profile/ucsf.psychiatry.bioinformatics.core/vizzes). The 3D structure (PDB: 6J8E) was visualized in PyMOL (The PyMOL Molecular Graphics System, Version 3.0 Schrödinger, LLC). The Na_v_1.2 structure comprises the human protein bound to the µ-conotoxin KIIIA and the subunit β2, forming the Na_v_1.2-beta2-KIIIA ternary complex. Protein segments embracing the 1–116, 285–313,442–739, 988–1191, and 1786–2005 amino acids remained undetermined (Pan et al. 2019) and could not be mapped onto the 3D structure for analysis. A diagram of the methodology applied to this work is shown in Fig. 1.

### Statistical Analyses

All data used for statistical analysis is shown as median with standard deviation (median +/- SD), with significant statistical differences being considered for p-value < 0,05. The Fisher’s exact test was performed to compare the aggregation of interspecies variant residues within the first cytoplasmatic linker versus all other residues. A Mann-Whitney U test was used for the comparison of the conservation scores between the interspecies variant residues and all other residues of the protein. These statistical analyses were performed using the Prism 8 software (GraphPad).

## Results

### Interspecies Comparison of Na_v_1.2 Amino Acid Sequence

**Fig. 2:**
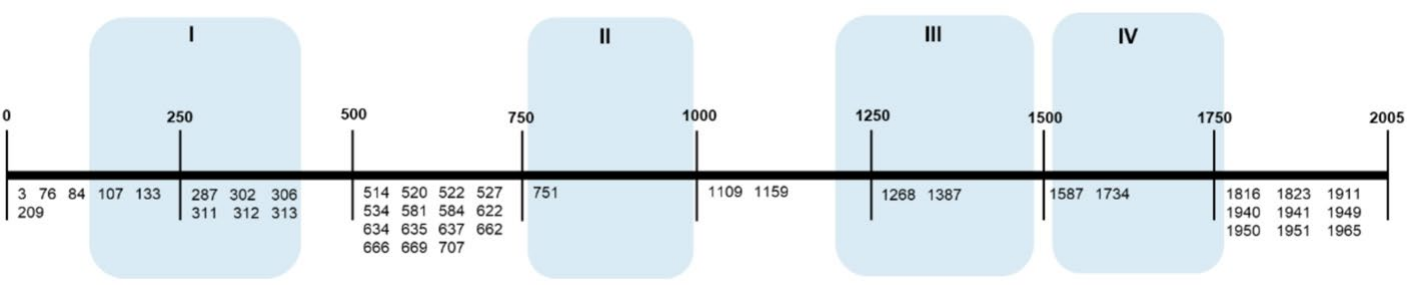
Schematic representation of the distribution of inter-species differences. Blue boxes represent the four domains of the Nav1.2 protein and numbers indicate a variable position between species

The alignment of Na_v_1.2 amino acid sequence between humans and other mammalian species revealed that 44 changes were seen between humans and non-human mammalian sequences, with only four amino acid positions (84, 313, 584 and 1734) distinct between humans and primates (Supplementary file 1). A highly conserved sequence block was observed between positions 760 to 1500, corresponding to the domains II and III of the protein (Fig. 2). Only position 1734 was shown to differ between humans and the other species: while humans have an Asp in this position, other species have a Glu at the same amino acid position.

Given the visual clustering of variants near the first cytoplasmatic linker (Fig. 2), we evaluated if this region is significantly enriched for interspecies differences. To do so, the number of interspecies variants inside this linker was compared to the number of variants in the rest of the protein using a Fisher’s exact test. In total, 17 variants were observed at the first cytoplasmic linker and 24 outside this region, indicating that the number of interspecies differences located at the first cytoplasmatic linker is significantly higher (p-value = 0,0003) than would be expected if no clustering existed.

**Fig. 3:**
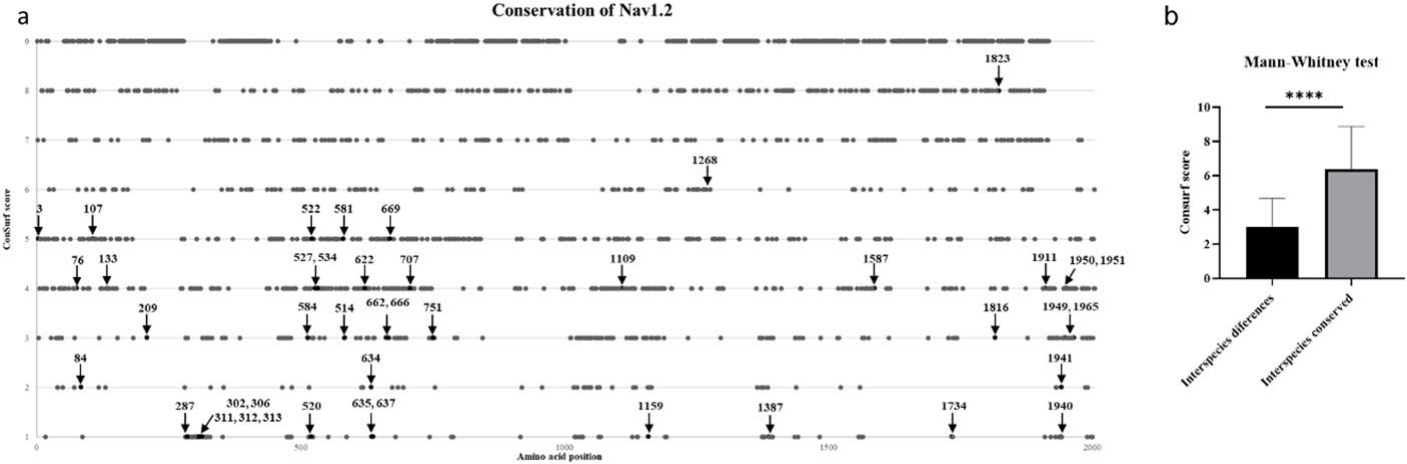
Conservation profile of the Na_v_1.2 protein. **a** Distribution of the conservation scores extracted from the ConSurf server. Black circles represent the residues that differ between species, while grey circles represent the conserved residues between humans and other mammalian species. **b** Mann-Whitney test of conservation scores of Na_v_1.2. Data are represented as mean ± SD, **** p<0.0001

We next evaluated the Grantham scores of these substitutions and a significant degree of evolutionary conservation across species was observed. The scores categorize 45.5% of the substitutions as being conservative, 34.1% as moderately conservative, 13.6% as moderately radical and only 6.8% as radical. Finally, we accessed the conservation of the Na_v_1.2 protein using the conservation scores calculated by ConSurf. The distribution of these scores across the protein is shown in Fig. 3. Statistical analysis revealed that the amino acid residues found across distinct species here analysed are significantly less conserved than the other residues of the protein (Fig. 3b). These results collectively reveal that the Na_v_1.2 retains an important level of conservation across different species due to its functional relevance.

### Intraspecies Comparison of Na_v_1.2 Sequences

**Fig. 4:**
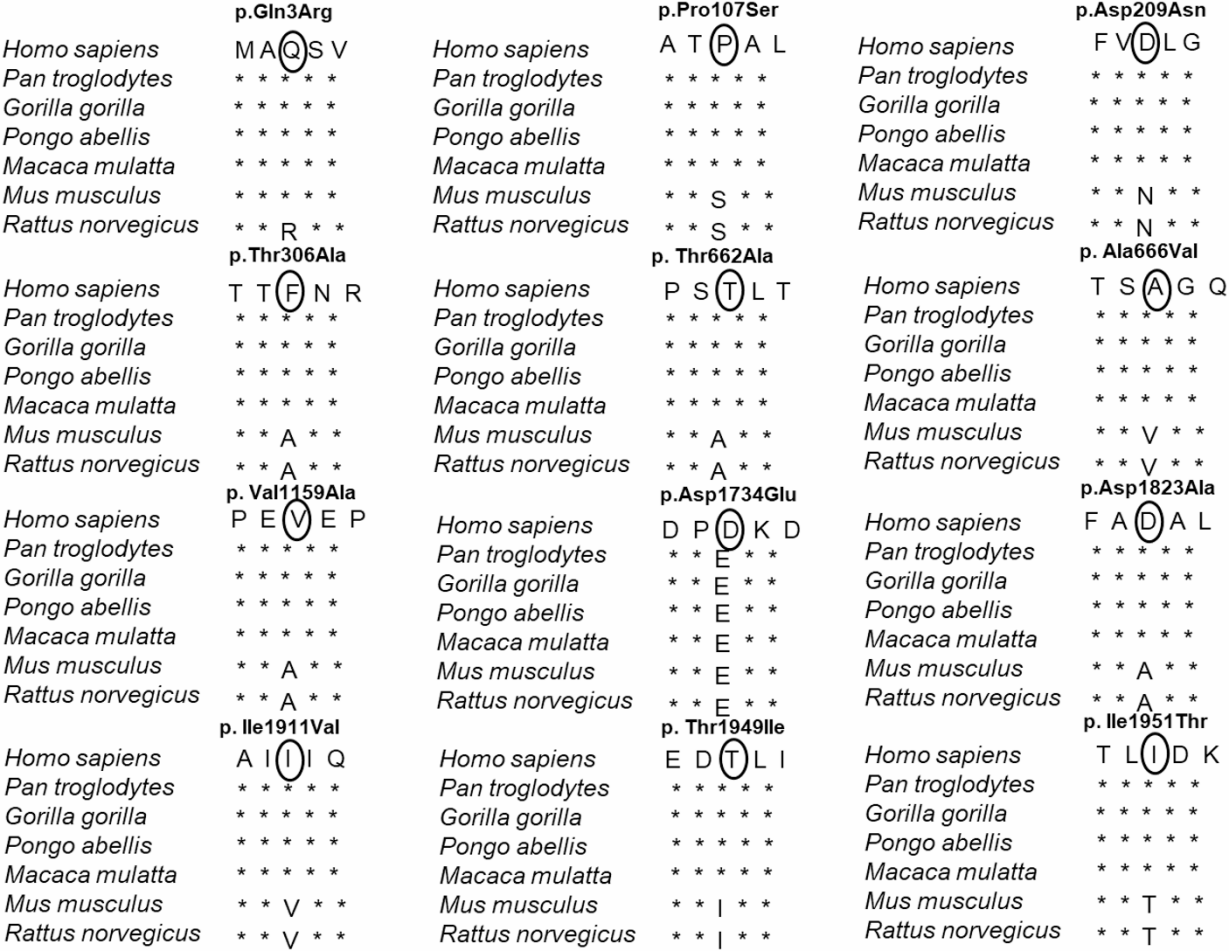
Partial alignment of human variants annotated at gnomAD and observed in the sequence of non-human mammals

The total set of variants found at gnomAD, which correspond to intraspecific variability was cross-referenced with the orthologous Na_v_1.2 amino acid sequences analysed in this study. In 12 amino acid residues, the human derived allele matches the native sequence of the non-human sequence (Fig. 4). In almost all cases, the human derived allele corresponds to the sequence of the two rodent species analysed. In one instance (p.Asp1734Glu), the human derived allele seems to be the reappearance of the ancestral amino acid observed in all non-human and rodent species studies.

Two of these residues are classified in gnomAD as “Conflicting interpretations of pathogenicity” (p.Thr662Ala; p.Asp1823Ala), and six of them were classified as “Uncertain significance” (p.Gln3Arg; p.Pro107Ser; p.Val1159Ala; p.Asp1823Ala; p.Ile1911Val; p.Thr1949Ile; p.Ile1951Thr). The identification of these variants, particularly those with conflicting interpretations of pathogenicity and uncertain significance, reveals the complexity of genetic variation and the need of functional studies to clarify their impact given the fact that they are the native allele in some mammalian species.

### Distribution of Variants in the Na_v_1.2 Structure

**Fig. 5:**
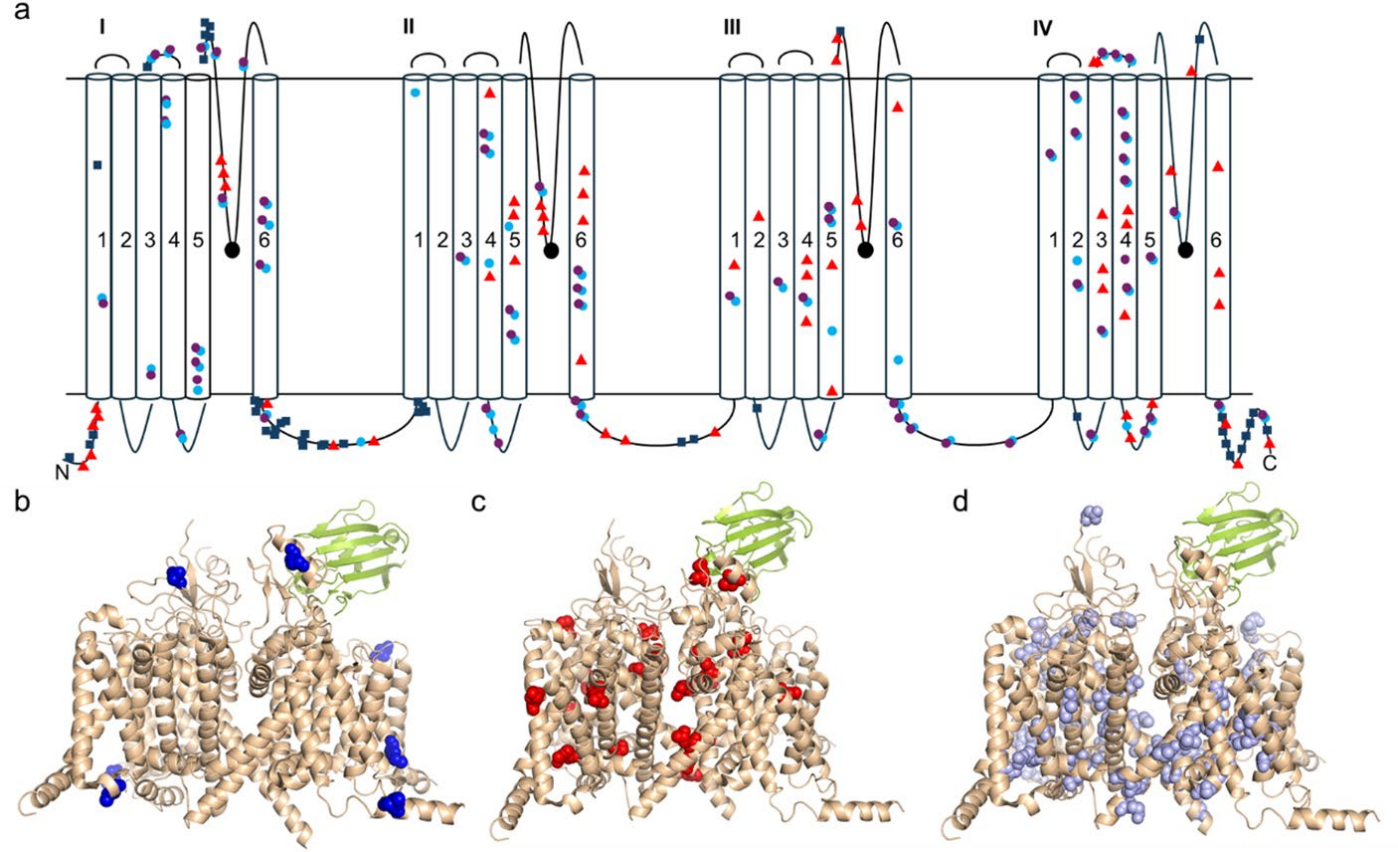
Structure of the Nav1.2 protein. **a** 2D structure of Nav1.2. Each symbol represents a different variant. Dark blue squares represent variants that differ between humans and other species. Red triangles represent variants associated with ASD (taken from Zhou et al. 2022 and from Sanders et al. 2018). Blue and light blue dots represent variants associated with DEE and SeLNE taken from the ClinVar database, respectively. **b**, **c**, **d** 3D representation of the human Nav1.2-beta2-KIIIA ternary complex (PDB: 6J8E). Colored in green is the β2 subunit, and in beige is the human Nav1.2 protein. Each sphere represents a different variant. **b** Dark blue spheres: interspecific variation. **c** Red spheres: ASD variants. **d** Light blue spheres: DEE/SeLNE variants

We next compared the distribution of variants observed in the comparison of species with the DEE, SeLNE and ASD-associated variants in the structure of the protein. (Fig. 5**)**. DEE/SeLNE-associated variants tend to aggregate in the S4-S5 segments of the domains and ASD variants in the first pore loop (Fig. 5a) as previously reported in the literature (Ben-Shalom et al. 2017; Sanders et al. 2018; Wolff et al. 2017). The 3D structure of Na_v_1.2 revealed a distinct pattern of distribution of variants between species compared to those associated with human disease (Fig. 5b–d). In fact, residues that differ between humans and the other species seem to locate at the outer region of the protein (Fig. 5b), whereas those linked to human disease (namely DEE/SeLNE and ASD) were observed buried within the protein (Fig. 5c–d).

## Discussion

Identifying human genetic variation and its impact on disease risk is an important step for personalized medicine, using naturally occurring variants to understand the phenotypic outcome of missense genetic variation (Bomba et al. 2017). Over the years, several studies have tried to categorize genetic variation and its impact on human health (Lek et al. 2016; Dewey et al. 2016; Van Hout et al. 2020; Taliun et al. 2021; Sun et al. 2024) offering a chance to estimate the frequency of rare variants, and improving the power of gene discovery for both common and rare diseases.

The assessment of the impact of deleterious variants can be done by understanding the evolutionary history of a protein. To understand the evolutionary history of Na_V_1.2 we compared the protein sequence between humans and six other mammals. A total of 44 differences were observed which indicates a high degree of selective constraints in the evolution of the protein, highlighting its functional importance. These differences were found at the N- and C-terminal regions, but preferentially in the first cytoplasmatic linker between the first and second domains and at the outer region of the 3D structure of the protein (Fig. 5a).

In contrast, a high proportion of disease-associated variants was observed at the core of the protein, where they are expected to result in great disturbances of the internal structural networks. Previous data have also revealed that the S4-S5 region in the voltage sensing domain, the intracellular N- and C-terminal regions, and the pore loops around the ion selectivity filter are more prone to deleterious variants, and that at the S4-S5 transmembrane segments are more often associated with SeLNE while mutations in the pore-loops are usually associated with ASD (Sanders et al. 2018).

The S4 segment of the Nav1.2 channel is a voltage-sensing region (de Lera Ruiz and Kraus 2015), while the S5 segment contributes to the pore-forming domain essential for ion conductance (Lipkind and Fozzard 2008). The S4-S5 linker region plays a critical role in the channel’s gating mechanisms, controlling the transitions between activation and inactivation states (Catterall 2017). Mutations within the S4-S5 linker have been associated with gating abnormalities that lead to gain-of-function effects (Scalmani et al. 2006; Ogiwara et al. 2009; Lauxmann et al. 2013; Schwarz et al. 2016), characterized by channel overactivity and enhanced excitability of glutamatergic neurons.

Loss-of function variants in the pore forming region S5-S6 segments are frequently associated with ASD (Tavassoli et al. 2014; Sanders et al. 2018; Wang et al. 2021; Zhou et al. 2022). These variants can impact the selectivity of the channel, reduce conductance, or increase inactivation, leading to impaired synaptic development and disruptions in the formation of cortical circuits. Dysfunction in Nav1.2 during early brain development can interfere with the maturation of neural circuits involved in cognition and social behavior, contributing to ASD phenotypes (Spratt et al. 2019).

From a therapeutic perspective, sodium channels are potential targets for drug treatments, and small molecules can be created to either increase or decrease the excitability of these channels, thus providing possible treatments (Sanders et al. 2018; Goodchild et al. 2024). Most therapies for Na_v_1.2-associated epilepsy (Li et al. 2024) use molecules that bind to different biophysical states of the channel, such as blocking the fast inactivation state of the channel or acting on the slow activation state (Pal et al. 2021).

Important advancements in this field can be achieved by understanding the evolutionary and pathologic mutational and structural dynamics of the channel Therefore, this work intends to contribute to a better understanding of the relevant structural elements of the Na_v_1.2 and may guide future research into their involvement into neurodevelopmental disorders.

## Supplementary Information

Below is the link to the electronic supplementary material.

Supplementary Material 1

## Data Availability

Data is provided within the manuscript or supplementary information files.
